# Stellate Ganglion Block Reduces Anxiety Symptoms by Half: A Case Series of 285 Patients

**DOI:** 10.3390/jpm13060958

**Published:** 2023-06-06

**Authors:** James H. Lynch, Sean W. Mulvaney, Craig J. Bryan, David Hernandez

**Affiliations:** 1 The Stellate Institute, 116 Defense Highway, Suite 203, Annapolis, MD 21401, USA; 2 College of Medicine, The Ohio State University, Columbus, OH 43210, USA; 3 The Retreat at Sheppard Pratt, 6501 N Charles Street, Baltimore, MD 21204, USA

**Keywords:** stellate ganglion block, anxiety, generalized anxiety disorder, novel therapeutics, sympathetic nervous system

## Abstract

The stellate ganglion block (SGB) procedure has been used successfully for over twelve years to treat thousands of patients suffering from posttraumatic stress disorder (PTSD). Level 1b evidence supports this use of SGB, but no studies to date have reported specifically on anxiety symptom improvements following SGB. We collected Generalized Anxiety Disorder questionnaire (GAD-7) scores pre-procedure and at 1-week and 1-month post-procedure from 285 patients. The mean baseline GAD-7 score of 15.9 (indicating severe anxiety) declined significantly following SGB treatment. Changes in GAD-7 scores ≥ 4 were considered clinically meaningful. From baseline to 1 week, the GAD-7 scores dropped by 9.0 points (95% CI = 8.3–9.7, *p* < 0.001, d = 1.8), with 211 (79.6%) patients demonstrating clinically meaningful improvement. Furthermore, from baseline to 1 month, the GAD-7 scores dropped by 8.3 points (95% CI = 7.6–9.0, *p* < 0.001, d = 1.7), with 200 (75.5%) patients demonstrating clinically meaningful improvement. The stellate ganglion block treatment resulted in a decrease of GAD-7 scores of over twice the minimal clinically important difference in treating anxiety for at least 1 month following SGB. Given the results from this retrospective observational study, larger prospective studies should be conducted to determine the effects of SGB treatment as a novel therapeutic treatment for generalized anxiety disorder and other anxiety disorders.

## 1. Introduction

Anxiety disorders are common and affect a large proportion of the US population with a significant impact on quality of life and functioning [1]. There is a 34% lifetime prevalence of anxiety disorders in the US. Additionally, anxiety disorders are on the rise among adult Americans under the age of 50 [2]. Specifically, the lifetime prevalence of anxiety disorders is higher in women than men (40% and 26%, respectively) [3]. Common anxiety disorders include generalized anxiety disorder, social anxiety disorder, and panic disorder with or without agoraphobia. These disorders are associated with significant impairments in social and occupational functioning, increased healthcare utilization, and overall reduced quality of life [1]. Anxiety disorders are responsible for an estimated annual global burden in excess of 26 million years lived with disability, and result in over $4 billion in workplace costs due to an average of 4.6 days of lost work per month [4,5].

While there is no single consensus guideline for the treatment of anxiety disorders, first-line treatments currently include pharmacotherapy and psychotherapy. Both selective serotonin reuptake inhibitors and serotonin–norepinephrine reuptake inhibitors are considered first-line pharmacotherapy treatments for anxiety disorders [1]. Second and third-line medications include benzodiazepines, tricyclics, azapirones, antihistamines, anticonvulsants, and beta-blockers. For treatment-refractory and/or comorbid conditions, monoamine oxidase inhibitors and atypical antipsychotics can be used. Moreover, cognitive behavioral therapy is recommended as the first-line psychotherapy for anxiety disorders [6]. For patients who do not demonstrate an adequate response to CBT or those who have limited access to psychotherapy, several complementary and integrative approaches can be prescribed. These include exercise, yoga, and mindfulness-based stress reduction.

Despite efficacious psychopharmacologic and psychotherapeutic interventions, many patients remain symptomatic [1]. There are several real-world challenges to treating anxiety disorders. Importantly, there is limited access to effective psychotherapy treatments, particularly in low-income and rural areas [7,8]. Comorbid conditions, such as substance use disorders, add complexity to treatment and often lead to a lower response to treatment [9]. There can also be adverse effects to medications, such as palpitations, jitteriness, nausea, drowsiness, or insomnia, which can be problematic for individuals receiving pharmacotherapy [1]. Treatment resistance is another major challenge. Only 45% to 65% of patients respond to initial treatment with either psychotherapy or pharmacotherapy [10]. Remission from anxiety disorders tends to be even lower from approximately 40% to 51% [11]. Overall, these challenges highlight the need for novel therapeutics to reduce the impact of these disorders on individuals and society.

Stellate ganglion block (SGB) is a procedure in which an injection of a long-acting local anesthetic, using ultrasound guidance, is made in the side of the neck around the main nerve that controls the sympathetic nervous system. This nerve, known as the cervical sympathetic chain, is a two-way conduit, and connects the brain’s fight or flight response to the rest of the body. SGB has been used safely to treat sympathetically-mediated pain syndromes and other autonomic disorders for over 100 years. [12,13,14]. By blocking or “turning off” the traffic in the cervical sympathetic chain, it is believed that the parts of the brain that control the fight or flight response are allowed to reset, resulting in long-term relief of symptoms from conditions such as posttraumatic stress disorder. The SGB takes less than 15 min to perform, and benefits are seen in as little as 30 min, with few to no long-term side effects.

Multiple peer-reviewed medical studies show that SGB results in a significant long-term improvement in chronic anxiety symptoms associated with posttraumatic stress disorder, primarily through targeting dysfunction of the autonomic nervous system. Lebovitz et al. first described the use of stellate ganglion block (SGB) to treat PTSD in 1990 [15]. Over the past 12 years, stellate ganglion block has been used successfully to treat thousands of patients with significant symptoms of posttraumatic stress disorder. There have been over 18 original studies published in the peer-reviewed literature which support the use of SGB for this indication [15,16,17,18,19,20,21,22,23,24,25,26,27,28,29,30,31,32].

Currently employed in practice, the established benefit of SGB for PTSD symptoms was the basis for our inquiry of anxiety as a separate indication for SGB therapy. Based on extensive anecdotal experience using stellate ganglion block to treat thousands of trauma survivors over the past 12 years, we suspected that, in addition to PTSD, SGB may provide benefits for anxiety disorders. This seemed plausible due to the significant symptom overlap between “trauma and stressor-related disorders” and anxiety disorders [33,34].

## 2. Materials and Methods

The Generalized Anxiety Disorder (GAD-7) questionnaire is a standardized tool used to assess anxiety symptoms. In addition to our usual screening using the PCL-5 self-reported questionnaire for PTSD, we added the GAD-7 questionnaire to the routine assessment process in our clinic. Study protocols were reviewed and approved by The Institute of Regenerative and Cellular Medicine, Santa Monica, CA Institutional Review Board (Protocol number: SI-SGB-001). The participants provided their written informed consent after a detailed discussion of the risks and benefits was conducted in person by one of the two performing physicians, JL or SM.

All stellate ganglion block treatments were conducted at The Stellate Institute in Annapolis, Maryland, USA by either one of the authors, JL or SM. All SGBs were performed under ultrasound guidance using a high-frequency linear probe with subjects lying supine with their heads rotated slightly away from the side of the injection. During treatments, standard sterile techniques were utilized. An anterolateral approach was used after carefully scanning each subject’s neck anatomy with Doppler imaging to identify common structures and vascular anomalies. Then, using a 2-inch 25-gauge needle, the neck was accessed with the needle in long axis to the ultrasound transducer (“in-plane” approach). The needle was visualized under real-time ultrasound guidance through the sternocleidomastoid muscle continuing just ventral to the tip of the anterior tubercle of C6 until the needle tip just penetrated the ventral fascia of longus coli, just medial to the longus capitis muscle and dorsal to the common carotid artery. Slowly over a minute, seven milliliters of 0.5% ropivacaine was injected in 0.5 mL aliquots (to mitigate the risk associated with potential intravascular injection). This same technique was then utilized at the C4 level with a volume of two milliliters of the same injectate. After remaining supine for five minutes, the subject was examined in a seated position by two independent clinicians for the presence of Horner’s syndrome findings, which were then scored (on a six-point scale, which has been previously described) to confirm that the SGB was successful in blocking sympathetic activity on the treated side [35].

For bilateral stellate ganglion blocks, the contralateral side was treated at least 12 h after the initial SGB for safety reasons as per our usual protocol. This is to completely avoid the extremely rare but potential airway compromise from bilateral vocal cord paresis if SGBs were performed on both sides at the same time.

### 2.1. Data Collection

Stellate ganglion block therapy at our center has been performed primarily for the treatment of PTSD symptoms. Beginning in March 2021, the GAD-7 questionnaires were completed by all of our center’s SGB patients in-person on paper or via email sent to our secure clinic email. The encrypted email system is the same system used for collecting routine patient information forms. The complete follow-up GAD-7 questionnaires (at one week and one month follow-ups) were collected from 285 patients by utilizing one of two following methods: by clinic email using the system we use for routine email follow-up or in-person. All data was collected at one location: our office, The Stellate Institute, 116 Defense Highway, Suite 203, Annapolis, MD 21401, telephone number—(410) 505-0530. The individual patient information was kept in the respective encrypted electronic medical records (EMRs) (AthenaOne) for routine medical follow-up and for assessment of the SGB therapy. Using non-identifying patient numbers (generated by the AthenaOne EMR software), a separate spreadsheet (on a secure medical computer) was used to compile the GAD-7 information (at baseline, one-week, and one-month post-treatment), as well as the dates of treatment, and the patient demographic information (sex, age).

### 2.2. Data Analysis

To test the pre–post change in the GAD-7 scores, we used mixed-effects modeling with a random intercept, random slope, and autoregressive covariance matrix. Time was entered as a categorical predictor (i.e., baseline, 1 week, 1 month). To determine if the change in GAD-7 scores differed between patients receiving one-sided versus bilateral injections, we used mixed-effects modeling with time, number of sides (one versus two), and the interaction of time and number of sides entered as predictors. Standardized mean differences (Cohen’s d) and minimum clinically important differences (MCID) were used to evaluate the postbaseline change in the GAD-7 scores. The MCID was calculated using a distribution-based approach [36], with GAD-7 change scores ≥ 4—approximately one standard deviation of the baseline mean score—set as the MCID threshold [37].

## 3. Results

**Figure 1:**
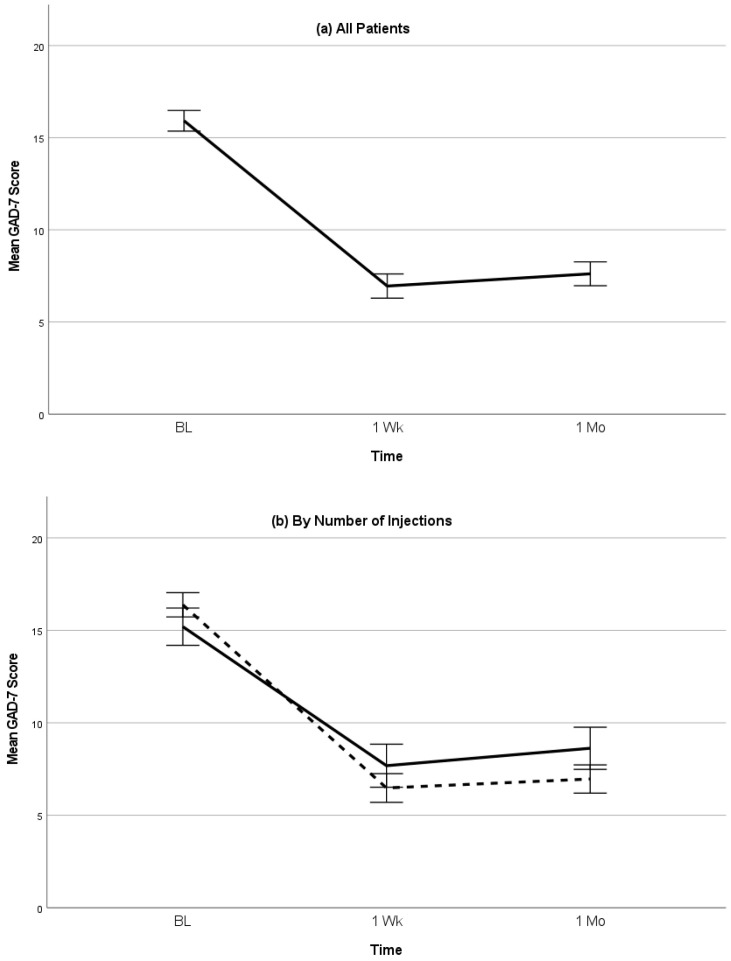
(**a**) Mean GAD-7 scores (with 95% confidence intervals) following SGB injection for all patients. (**b**) Mean GAD-7 scores (with 95% confidence intervals) following SGB injection involving either one-sided injection (solid line) or bilateral injection (dashed line).

Patients included 128 men (48.3%) and 137 women (51.7%) ranging in age from 19 to 81 years (M = 45.3, SD = 12.7). Most patients (*n* = 161, 60.8%) received bilateral injections. The mean baseline GAD-7 score was 15.9 (SD = 4.6), indicating severe anxiety [38]. The GAD-7 scores significantly declined over time (F(1, 488) = 411.2, *p* < 0.001; see Figure 1a). From baseline to 1 week, the GAD-7 scores dropped by 9.0 points (95% CI = 8.3–9.7, *p* < 0.001, d = 1.8), with 211 (79.6%) patients demonstrating clinically meaningful improvements. Additionally, from baseline to 1 month, the GAD-7 scores dropped by 8.3 points (95% CI = 7.6–9.0, *p* < 0.001, d = 1.7), with 200 (75.5%) patients demonstrating clinically meaningful improvements.

The GAD-7 score reductions were larger among patients who received bilateral versus one-sided injections (F(2, 487) = 9.7, *p* < 0.001; Figure 1b). Among patients receiving one-sided injections, the GAD-7 scores dropped from baseline to 1 week by 7.5 points (95% CI = 6.3–8.8, *p* < 0.001, d = 1.4) and by 6.6 points (95% CI = 5.4–7.8, *p* < 0.001, d = 1.2) from baseline to 1 month. Furthermore, among patients receiving bilateral injections, the GAD-7 scores dropped from baseline to 1 week by 9.9 points (95% CI = 9.1–10.7, *p* < 0.001, d = 2.2) and 9.4 points (9.1–10.7, *p* < 0.001, d = 2.1) from baseline to 1 month. At 1 week, 76 (73.1%) patients receiving a one-sided injection and 135 (83.9%) patients receiving a bilateral injection demonstrated clinically meaningful improvements, and, at the 1 month follow-up, 70 (67.3%) patients receiving the one-sided injection and 130 (80.7%) patients receiving the bilateral injection demonstrated clinically meaningful improvements.

## 4. Discussion

While this is the first report to demonstrate the effect of SGB on anxiety symptoms, there are several limitations to this study. As a retrospective analysis of anxiety symptoms, the lack of a control group limits the conclusions which can be drawn. Most of the subjects treated with SGB were receiving concomitant psychotherapy, pharmacotherapy, or a combination of both. These other variables were not controlled for or considered to be exclusion criteria. In fact, though, a real-world clinical study such as this one may be useful precisely because we can assess the effect that SGB had on anxiety symptoms when *added to* the subjects’ current therapies.

The overlap of PTSD and anxiety symptoms is common and, therefore, allows for the extrapolation of the same mechanism to work for an alternate diagnosis, such as generalized anxiety disorder (GAD). SGB has been described as a *therapy catalyst* which increases compliance and promotes more effective gains from talk therapy by reducing physiologic hyperarousal symptoms [39]. This effect is critical in understanding the value SGB plays in *complementing* other therapies, rather than being considered as a second- or third-line treatment because hyperarousal symptoms serve as a significant barrier to effective talk therapy and exposure therapy in many anxiety sufferers [40,41].

Understanding the mechanism of action for SGB is critical to appreciate how this treatment modality relieves anxiety symptoms. Some symptoms queried on the GAD-7 questionnaire such as “feeling nervous, anxious, or on edge;” “trouble relaxing;” or “becoming easily annoyed or irritable” are rooted in the physiologic expression of the sympathetic nervous system, which may be inappropriately elevated in those suffering from anxiety disorders. The cervical sympathetic chain in the neck carries sympathetic nervous system signals in both directions—from the brain to the body and from the body to the brain. Similar to other neurologic hyperarousal conditions, such as complex regional pain syndrome (CRPS), anxiety symptoms may be stuck in an elevated state due to this two-way conduit positively feeding back on itself. This results in a loop which may be refractory to interruption because it resides in the autonomic nervous system, which functions beneath our consciousness. The automatic nature of the fight or flight system is absolutely critical for our survival because the speed at which we would otherwise respond to threats would be insufficient to survive in many cases. Therefore, if there is specific dysfunction in the autonomic nervous system which may be refractory to standard therapies, precisely targeting the anatomic structures which govern it may provide a precise mechanism to treat the dysfunction. Turning off this “dysfunctional circuit” by temporarily numbing the cervical sympathetic chain with a long-acting anesthetic (e.g., ropivacaine) disrupts the loop between the brain and the body and allows the circuit to reset itself. This mechanism for the treatment of neurologic dysfunction is not new. In fact, this same principle of utilizing nerve blocks to reset nerve signals has been a validated treatment modality in pain medicine for many years [42,43,44].

## 5. Conclusions

Overall, stellate ganglion block treatment resulted in a decrease of GAD-7 scores of over twice the minimal clinically important difference for treating anxiety. This effect was sustained for at least 1 month following the SGB injection. This study also demonstrated the first observation of a superior benefit from bilateral over unilateral SGBs, which has important implications in clinical practice. Thus, SGB may be explored as a safe, rational therapeutic option when considering treatment for generalized anxiety disorder (GAD) symptoms or other anxiety disorders (e.g., panic disorder, social anxiety disorder, specific phobias). Similar to the mechanism of action used to explain the benefit of treatment for PTSD symptoms, the effects on anxiety symptoms may be explained as a result of directly resetting the sympathetic nervous system by temporarily blocking the conduction of the cervical sympathetic chain. Further prospective research is warranted to explore the effects of SGB on anxiety symptoms.

## Data Availability

All clinical data is contained within the manuscript.
